# Health-related quality of life in prisoners with attention-deficit hyperactivity disorder and head injury

**DOI:** 10.1186/s12888-018-1785-9

**Published:** 2018-06-22

**Authors:** Susan Young, Rafael A. González, Moshe Fridman, Paul Hodgkins, Keira Kim, Gisli H. Gudjonsson

**Affiliations:** 1 Psychology Services Limited, PO Box 1735, Croydon, CR97AE UK; 2 Reykjavik University, Reykjavik, Iceland; 3 East London NHS Foundation Trust, East London, UK; 4 AMF Consulting Inc., Los Angeles, CA USA; 5 Sage Therapeutics, Cambridge, MA USA; 6 Independent Medical Writer, San Diego, CA USA; 7 Institute of Psychiatry, Psychology and Neuroscience, King’s College London, London, UK

**Keywords:** Attention deficit/hyperactivity disorder, Head injury, Traumatic brain injury, Prison population, Health-related quality of life, Co-morbidity

## Abstract

**Background:**

Attention-deficit hyperactivity disorder (ADHD) and head injury (including traumatic brain injury (TBI)) manifest in high levels across prison samples and guidance from the National Institute for Health and Care Excellence notes that people with acquired brain injury may have increased prevalence of ADHD. We aimed to examine the association of ADHD with TBI and the impact of the association upon health-related quality of life (HRQoL) and service use among imprisoned adults.

**Methods:**

An observational study was performed in 2011–2013, at Porterfield Prison, Inverness, United Kingdom (UK). The all male sample included 390 adult prison inmates with capacity to consent and no history of moderate or severe intellectual disability. Head injury was measured with a series of self-reported questions, addressing history of hits to the head: frequency, severity, loss of consciousness (LOC), and sequelae. Participants were interviewed using the Diagnostic Interview for ADHD in Adults 2.0. The Health Utilities Index Mark 3 was used to measure health status, and to calculate attribute specific HRQoL and Quality-Adjusted Life Year (QALY) scores.

**Results:**

72% of prisoners sampled reported at least one head injury in their lifetime. Among those, 70% of head injuries occurred before age 16 and 70% experienced LOC. Prisoners with ADHD were nearly twice more likely to have TBI. Prisoners with ADHD-only and ADHD with co-morbid TBI had significantly lower scores in several HRQoL attributes, compared with TBI only or the absence of either condition. Adjusted logistic regression models indicated an average reduction of 0.20 QALYs in inmates with ADHD-only and 0.30 QALY loss in those with ADHD with co-morbid TBI compared with inmates with neither condition.

**Conclusions:**

There is a robust association between ADHD and TBI, and ADHD with co-morbid TBI confers significantly greater impairment in terms of HRQoL. Managing the short and long-term consequences of TBI is essential to improving care for prisoners and to addressing the criminogenic factors related to them.

## Background

Despite the high prevalence of both attention-deficit hyperactivity disorder (ADHD; 26%) [1] and head injury (including traumatic brain injury (TBI; 60%)) [2] in the prison populations, little is known about their association with each other and their impact on health-related quality of life (HRQoL) measures. The recently revised National Institute for Health and Care Excellence guideline on the recognition of ADHD (NICE guideline NG87) noted that people with acquired brain injury may have increased prevalence of ADHD compared with the general population [3]. Hence the present study is topical in investigating quality of life in offenders with ADHD and head injury.

ADHD is a childhood onset neurodevelopmental disorder, characterised by a persistent pattern of inattention and/or hyperactivity-impulsivity [4]. ADHD is associated with executive functioning deficits [5] and combined with conduct problems is associated with an increased risk for adult criminality [6]. Compared with world-wide prevalence rates (2.5% adults [7], 5.3% children [8]), there is a five-fold increase of ADHD among youth prisoners (< 18 years) and a ten-fold increase of ADHD among adult prisoners (> 18 years) [1]. A systematic review and meta-analysis study indicated that among incarcerated adults with ADHD there is an increased rate of co-morbid psychiatric disorders (29% conduct disorder (CD), 74% substance use disorder, 66% mood disorder, 55% depressive disorder, 68% anxiety disorder, and 60% personality disorders) [9]. A study of 1.92 million individuals in Denmark demonstrated a significant increase in the mortality rate among people with ADHD compared to those without ADHD (*p* < 0.0001), with accidents being the leading cause of death [10].

Prevalence rates for head injury and TBI vary depending on the source of information, definition, the occurrence of loss of consciousness (LOC), and/or loss of memory. A meta-analysis indicated that 60% of the overall offender population is reported to have TBI with LOC [2], which is significantly higher than the general population estimates of 12% in adults [11] and 24% in adolescents [12]. A recent systematic review reported that between 16.5 to 72.1% of incarcerated youths have TBI [13]. Two studies indicated that prisoners commonly report a history of repeated head trauma incidents [14, 15]. A population study based on Swedish registry data reported that individuals with TBIs are at significant risk of violent crime [16]. Furthermore, prisoners with TBI were reported to be significantly younger at the time of their first offence, compared with prisoners without TBI [17].

The possible association between ADHD and head injuries is complex. Difficulties with the ability to self-regulate have been described as an executive function that is central to ADHD symptoms [18], which may be an antecedent for impulsive behaviour or aggression [19]. Therefore, having ADHD may increase the risk of head injuries [10, 20]. Additionally, there is some evidence to suggest that brain injuries in school-age children may lead to the development of ADHD later in childhood [20, 21]. Furthermore, ADHD and TBI share similar risk factors, such as low socioeconomic status and history of risk-taking behaviours [22, 23] and similar symptoms. TBI is usually accompanied by attention deficits, frontal-executive compromise, and self-regulation difficulties [24]. Because TBI is often the outcome of certain risk taking behaviours, confounding effects from co-existing disorders such as ADHD and conduct problems may play an important role in associations.

In considering impairments and functional states that may be affected by disease, injury, and treatment, the Health Utilities Index Mark 3 (HUI3) is an effective tool to evaluate overall health status and quality of life outcomes [25, 26]. Results from HUI3 can be used to calculate attribute specific health-related quality of life (HRQoL) and quality-adjusted life years (QALY) scores, which represent the impact that health status has on quality of life and life span.

To the best of our knowledge this is the first study addressing the health status of adult prison inmates with ADHD and TBI using the HUI3. We hypothesize that: 1) there is a significant association between ADHD and TBI amongst prison inmates, and 2) prisoners with ADHD and co-morbid TBI have an increased likelihood of adverse health, intellectual ability, and quality of life outcomes, compared with those with ADHD only, TBI only, or neither.

## Methods

### Participants and sample selection

Following approval from the Scottish Prison Service Research Access and Ethics Committee and in accordance with the Declaration of Helsinki, prisoners were recruited by opportunity sampling from Porterfield Prison, Scotland, UK, over a period of 18 months in 2011–2013. Participants included 390 adult male prisoners who consented to participate. Those with moderate or severe learning difficulties, lack of fluency in the English language, and severe mental illness were excluded from participating.

Participants in the study were indirectly compensated. The study group deposited £20 per participant into a Prison Common Good Fund, which was managed by a group of prisoners. The fund was then used to purchase items for the common good of all prisoners to enhance prison life.

Prisoners who indicated interest attended an appointment with the researcher where they were given detailed (oral and written) information about the study and consent procedures. After obtaining consent, two psychology researcher assistants administered a comprehensive battery of measures, which took approximately 4 h to complete (usually split across 2 or 3 sessions). The researchers received comprehensive training to administer the Diagnostic Interview for ADHD in Adults 2.0 (DIVA-2) from the Maudsley Hospital Adult ADHD Service, which included observations and training on reaching diagnostic classification to ensure inter-rater reliability [27]. The researchers sat down with the participants throughout the sessions, administered the DIVA-2 and self-report questionnaires. Participants received assistance to do the latter, when required. Out of the 390 participants, 81 (20.8%) required assistance with reading the questionnaires. The request for assistance was most frequent among the 53 participants in the combined ADHD and co-morbid TBI group (41.5%), in contrast to 17.5% among the remaining 337 participants (Chi^2^ (*df* 1) = 8.20. *p <* 0.01).

### Measures

#### History of head injury

Head injury was measured via a self-reported questionnaire addressing history of hits to the head. The key question “Have you ever been hit in the head?” was followed by further questions detailing the injury and its consequences including: frequency, age of onset, occurrence of LOC, cause, related medical service use, and occurrence of related memory loss.

Definitions of TBI are often based on the occurrence of any LOC and on the extent of post-traumatic amnesia (PTA). Considering that head injury incidents were self-reported by prisoners, we aimed to make the identification of a likely TBI more robust. Recording the number of head injuries increased the threshold level and minimised false positives, and also helped to distinguish those most likely to have had any form of sequelae after their injuries.

#### Our strict definition of a likely TBI

We determined the lifetime prevalence of a likely TBI based upon the occurrence of the following:

1. At least one head injury **and**
2. Occurrence of LOC **and**
3. Any additional indicator of head injury severity (such as an LOC > 20 min, requiring surgery, and/or caused memory loss) **or**
4. Medical service utilisation related to the head injury (such as a visit to the doctor or Accident and Emergency (A&E) services).

Because head injury history was not entered into prisoners’ medical records, we were unable to formally apply the commonly used Mayo Classification System (MCS) [28]. But, by restricting our likely TBI cases to include only those with LOC (whilst also including a parameter of severity); all of our defined cases could be interpreted to be mild or moderate-severe by the MCS.

#### Health utilities index mark 3 (HUI3)

The HUI3 is a multi-attribute health status classification system that enables researchers to map levels of: vision, hearing, speech, ambulation, dexterity, emotion, cognition, and pain; using decision tables and coding algorithms, which can be represented in terms of attribute specific HRQoL scores [26]. The HRQoL scoring system provides utility scores ranging from 0.00 (dead) to 1.00 (perfect health), and meets the criteria for calculating Quality-Adjusted Life Years (QALY) [26]. The HUI health-based classification systems have been validated worldwide. In their paper [26] Horseman and colleagues direct readers to “the HUI web page at http://www.healthutilities.com and review the annotated references of articles from hundreds of studies worldwide for evidence of HUI validity (face validity, content validity, construct validity, convergent validity, discriminative validity, predictive validity), reliability and responsiveness.”

Prisoners’ were asked to answer HUI3 questions based upon their health status in the 4 weeks prior to the interview. The HUI3 composite score was used to calculate QALYs and was extrapolated to one year to represent the study health evaluation time frame.

#### ADHD diagnosis and CD screening

All participants underwent a comprehensive evaluation for ADHD and were interviewed using the Diagnostic Interview for ADHD in Adults 2.0 (DIVA-2) [29]. The DIVA-2 is a validated semi-structured clinical interview of ADHD in adults based on DSM-IVR criteria [4]. It is a clinical diagnostic tool and the associated website provides information about its validity (see www.eunetworkadultadhd.com/validation-of-diva-2-0/). Questions addressed their current and childhood (ages 5 to 12) presentation of ADHD symptoms and scope of impairment.

All participants were screened for conduct disorder using the CD scale of the Barkley Adult ADHD Rating Scale-IV (BAARS-IV) [30], which corresponds with DSM-5 criteria. Endorsement of three or more criteria indicated likelihood of the disorder.

#### Service use

Medical service utilization was obtained through inspection of prison medical records. Researchers recorded service use over the three months prior to the interview date, such as: visits to medical doctor, visits to accidents and emergency room (A&E), admission to hospital and length of stay, and surgeries. The authors chose to include three months of service for practical reasons; and additionally felt this time period fairly represented the service use of all prisoners given the variance in prison stays.

#### Intelligence

IQ was estimated using the vocabulary subtest of the Wechsler Abbreviated Scale of Intelligence (WASI) [31]. The vocabulary subtest is reported to be a robust predictor of general IQ regardless of brain injury [32].

#### Analytical strategy

Frequencies were reported for all categorical variables, and means (with standard deviations) for continuous variables. Student *t-*tests were used to contrast age and age of first injury by groups. Logistic regression models were fitted using ADHD as the exposure for any head injury, TBI, causes and consequences of head injury, and service utilisation. Ordered logistic regression was used to analyze number of head injuries reported.

Odds ratios (OR) were reported as indicators of the magnitude of associations in binary (e.g. presence or absence of head injury) and on the ordered categorical outcome (head injuries reported) models. Logistic regression analyses were repeated exclusively on those who reported their first injury before age 16. This analysis allowed us to examine whether a TBI occurring before age 16 may be associated with ADHD. We used age 16 because it represented the threshold for legal adulthood in the UK.

*Tobit* regressions were used in favour of traditional ordinary least-squares (OLS) regression models to estimate the relationship between inmate groups of ADHD only, TBI only, ADHD with co-morbid TBI, and groups having neither (reference group); and HUI3 HRQoL and QALY scores. The *Tobit* model is designed to estimate linear relationships between variables when there are ceiling or flooring effects on the outcome [33]. In our case, we considered the upper limit of 1.0 of the outcome variable as censored. Post-estimation linear combinations of coefficients allowed contrasting the ADHD with co-morbid TBI group with either ADHD only or TBI only group. Differences in estimated IQ were examined via linear regression models.

All models were adjusted for age at time of survey and for the presence of CD whenever necessary. We established the significance level at *p* ≤ 0.05 for all statistical tests. All analyses were performed using Stata version 13 (StataCorp) [34].

## Results

### Descriptive statistics

The all male sample was essentially Caucasian British (99.0%) with an average age of 30.3 years (sd 8.3). Mean ages for each of the groups were: 28.6 years (sd 7.9) for ADHD only, 32.3 years (sd 8.4) for TBI only, 27.9 years (sd 7.2) for ADHD with co-morbid TBI, and 30.2 years (sd 8.5) for neither ADHD nor TBI. Bonferroni post hoc analyses indicated a significantly younger age for the ADHD with co-morbid TBI group contrasted against the TBI only group.

### Head injury and TBI: Prevalence, history, and sequelae

Two hundred eighty participants (71.8%) reported having experienced at least one head injury in their lifetime, and 169 (43.3%) were considered to have a likely TBI according to our strict definition (see Table 1). Among those with at least one head injury, the mean age of first injury was 12.5 years (sd 6.7); approximately 70% of which suffered their first injury before age 16 and reported having experienced LOC (see Table 2).

**Table 1:** Head Injury, TBI, and IQ Among Inmates without ADHD and with ADHD

	All Inmates *N* = 390 n (%)	without ADHD *n* = 294 n (%^a^)	with ADHD *n* = 96 n (%^b^)	OR (95% CI)	*p*
ADHD prevalence		294 (75.4^c^)	96 (24.6^c^)		
Head injury frequency					
None	110 (28.2)	90 (30.6)	20 (20.8)		
1	64 (16.4)	57 (19.4)	7 (7.3)		
2 to 3	82 (21.0)	61 (20.8)	21 (21.9)		
4 to 6	63 (16.2)	39 (13.3)	24 (25.0)		
7 to 10	43 (11.0)	32 (10.9)	11 (11.5)		
11 or more	28 (7.2)	15 (5.1)	13 (13.5)	2.19 (1.44, 3.31)^d^	< 0.001
Any head injury	280 (71.8)	204 (69.4)	76 (79.2)	1.68 (0.97, 2.91)	*0.070*
Likely TBI	169 (43.3)	116 (39.5)	53 (55.2)	1.89 (1.19, 3.01)	*0.007*
IQ estimate, *mean [sd]*	*76.9 [14.4]*	*78.2 [13.9]*	*72.9 [15.3]*	t 3.10 (385)^e^	*0.002*

^a^Percentage of inmates without ADHD

^b^Percentage of inmates with ADHD

^c^Percentage of all inmates

^d^Derived from ordered *logit* model. Proportionality of odds was established using a chi-square test (*p* = 0.08)

^e^t distribution and degrees of freedom

**Table 2:** Head Injury History and Sequelae Among Inmates without ADHD and with ADHD

	Any head injury *n* = 280 n (%^a^)	without ADHD *n* = 204 n (%^b^)	with ADHD *n* = 76 n (%^c^)	OR (95% CI)	*p*
First injury age^d^, *mean yr [sd]*	*12.5 [6.7]*	*13.1 [7.1]*	*10.9 [4.9]*	t 2.45 (273)^e^	0.01
at < 16 yr	192 (69.8)	132 (65.4)	60 (82.2)	2.45 (1.26, 4.76)	0.008
at ≥16 yr	83 (30.2)	70 (34.7)	13 (17.8)	Referent	–
LOC	195 (69.6)	137 (67.2)	58 (76.3)	1.58 (0.86, 2.88)	0.14
LOC > 20 mins.	61 (21.8)	41 (20.1)	20 (26.3)	1.42 (0.77, 2.63)	0.26
Memory loss	65 (23.3)	43 (21.1)	22 (29.3)	1.55 (0.85, 2.83)	0.15
Caused by accident	121 (43.8)	93 (46.3)	28 (37.3)	1.45 (0.84, 2.49)	0.18
Caused by assault	155 (56.2)	108 (53.7)	47 (62.7)	Referent	–
Required doctor visit	220 (78.6)	155 (76.0)	65 (85.5)	1.87 (0.91, 3.82)	0.09
Required A&E visit	214 (76.4)	155 (76.0)	59 (77.6)	1.10 (0.59, 2.06)	0.77
Required surgery	54 (19.3)	38 (18.6)	16 (21.1)	1.16 (0.61, 2.24)	0.65

^a^Percentage of inmates with more than one head injury

^b^Percentage of inmates without ADHD

^c^Percentage of inmates with ADHD

^d^Age is based on data for 275 inmates, missing first injury age for 5 inmates

^e^t distribution and degrees of freedom

Among those who suffered at least one head injury; 79% required a visit to a medical doctor, 76% required a visit to accident and emergency (A&E) services, and 19.3% required surgery. Additionally, 23% reported experiencing either anterograde or retrograde memory loss. 44% of the head injuries were due to an accident, and 56% were the consequence of a violent assault (e.g. hit with bat) (see Table 2).

#### Head injury, TBI, and ADHD

24.6% of the prisoners met the criteria for a clinical diagnosis of ADHD. 79% of prisoners with ADHD and 69% of prisoners without ADHD reported at least one head injury (*p* = 0.07). Among prisoners reporting at least one head injury, prisoners with ADHD did not differ from those without ADHD in terms of cause, LOC, or sequelae (such as requiring emergency services, surgery, or experiencing memory loss) (see Table 2).

Using our strict definition of a likely TBI, there was a significant difference between prisoners with ADHD compared with those without ADHD. 55% of prisoners with ADHD had a likely TBI compared with 40% of those without ADHD (*p* < 0.01), with nearly twice the odds among those with ADHD to have experienced a TBI (see Table 1).

Among prisoners reporting head injuries, those with ADHD reported a significantly higher number of head injuries than those without ADHD (OR 2.19 (1.44, 3.31), *p* < 0.001). Notably, a significantly high proportion (82%) of those with ADHD and at least one head injury reported their first injury occurring before age 16 (OR 2.45 (1.26, 4.76), *p* < 0.001) (see Table 2).

Out of the total sample of 390, 153 (39.2%) met screening criteria for CD. There was a significant difference in CD screening diagnosis across the four groups: ADHD only; TBI only; ADHD/TBI; and remaining participants (Chi^2^ (*df* 3) = 43.6 *p <* 0.000). The rate was highest among the ADHD/TBI group (69.8%), followed by ADHD only (65.1%). The rate for TBI only was 29.3% and similar to the rate of 30.3% for none of the ADHD, TBI or combined conditions being met.

Analyses were repeated exclusively on those who reported their first injury before age 16. Although ADHD was associated with first injury occurring before age 16 it was no longer significant after adjustments were made for coexisting CD.

### Health status, service use, and estimated IQ

Prisoners with ADHD only, TBI only, and ADHD with co-morbid TBI were compared against those with neither in specific HRQoL attribute and QALY loss, service use, and estimated intelligence (see Table 3).

**Table 3:** Health Status, Service Use, and Estimated IQ of Prisoners with ADHD only, TBI only, and ADHD with co-morbid TBI Compared with Inmates with Neither

	ADHD only *n* = 43, 11.0%	TBI only *n* = 116, 29.7%	ADHD with co-morbid TBI *n* = 53, 13.6%
HUI3 HRQoL specific attribute scores^a^	beta coef. (se)	beta coef. (se)	beta coef. (se)
Vision	−0.04 (0.02)	−0.01 (0.01)	− 0.05 (0.02)**
Hearing	− 0.50 (0.30)	−0.09 (0.24)	− 0.57 (0.30)
Speech	−0.07 (0.07)	− 0.05 (0.05)	−0.10 (0.06)
Ambulation	−0.37 (0.40)	−0.48 (0.32)	− 0.81 (0.37)*
Dexterity	− 0.02 (0.16)	− 0.21 (0.12)	− 0.06 (0.15)
Emotion	−0.17 (0.07)*	− 0.03 (0.05)	−0.23 (0.06)***
Cognition	− 0.24 (0.07)**	− 0.06 (0.05)	−0.32 (0.07)***
Pain	− 0.09 (0.10)	−0.15 (0.07)*	− 0.23 (0.09)*
QALY derived by HUI3 composite score^*a,b,c*^	− 0.20 (0.06)**	− 0.05 (0.04)	−0.30 (0.05)***†
Service use (3 months)	OR (95%CI)	OR (95%CI)	OR (95%CI)
General Practitioner	1.09 (0.33, 5.60)	2.15 (0.75, 6.14)	1.09 (0.36, 3.32)
Physical Health Nurse	1.67 (0.45, 6.19)	0.94 (0.41, 2.13)	1.57 (0.48, 5.12)
Mental Health Nurse	1.59 (0.79, 3.22)	0.99 (0.59, 1.65)	3.08 (1.59, 5.98)**
Addiction Nurse	0.51 (0.23, 1.12)	1.13 (0.69, 1.85)	2.12 (1.10, 4.09)*
Estimated IQ, *mean (sd)*^*a*^	− 4.68 (2.48)	−1.62 (1.72)	−7.62 (2.34)**

Note: The referent is inmates with neither ADHD nor TBI, *n* = 178

^a^Models adjusted for age and CD

^b^HUI3 composite score was based on 362 inmates with complete HUI3 data (missing some specific attribute scores on 28 inmates)

^c^All *tobit* models account for censoring at the upper level of the outcome (QALY)

†Linear combination of coefficients for ADHD/TBI *v*. ADHD-only is not statistically significant; Linear combination of coefficients for ADHD/TBI *v*. TBI-only 0.25 (0.06), *p* < 0.001

*p < 0.05

**p < 0.01

***p < 0.001

Prisoners with ADHD-only had significantly poorer utility scores on emotion (*p* < 0.05) and cognition (*p* < 0.01) attributes. Those with ADHD with co-morbid TBI had the most impaired specific health attributes with significantly poorer scores on vision (*p* < 0.001), ambulation (*p* < 0.05), emotion (*p* < 0.001), cognition (*p* < 0.01) and pain (*p* < 0.05) attributes. Prisoners without ADHD or likely TBI had an average QALY of 0.72 (sd 0.26) and both the ADHD-only and ADHD with co-morbid TBI groups had significantly greater loss (− 0.2 and − 0.3, respectively) than those with neither.

Prisoners with ADHD with co-morbid TBI had a significantly higher number of visits to mental health nurses (*p* < 0.01) and addiction services (*p* < 0.05) compared with those with neither. Adjusted models for IQ demonstrate that prisoners with ADHD with co-morbid TBI had significantly lower intelligence scores compared to those with neither (*p* < 0.01) (Table 3). Because only the ADHD with co-morbid TBI group was significantly different from the referent in terms of IQ and service use, we made no further contrasts.

## Discussion

In the present study we set out to examine the potential association of ADHD with TBI in a UK prison sample and the impact that each has on health-related quality of life. Our hypotheses were supported. Our results indicate that prisoners with a clinical diagnosis of ADHD were significantly associated with our strict definition of a likely TBI; 55% were almost twice more likely to have TBI. Furthermore, our results indicate a high frequency of head injury at an early age; 82% of prisoners with ADHD with at least one head injury reported that their first injury occurred before age 16. In addition, the risk for adverse health, intellectual ability, and quality of life outcomes, appears greater among prisoners having ADHD with co-morbid TBI than for those with ADHD-only, TBI-only, or neither.

Our results are not consistent with another study addressing head injury among youth offenders [35]. There the authors reported that the proportion of youth offenders who were screened for ADHD and other neurodevelopmental disorders was not significantly higher amongst youth offenders with TBI, compared with those without TBI [35]. This apparent lack of an association between ADHD and TBI may be explained by having a small sample of juvenile offenders and/or by using different methods of assessing ADHD.

Our results are consistent, however, with findings from a recent prison study examining externalizing symptoms and injury severity [36]. Increased rates of externalizing behaviours were reported amongst young adult survivors of paediatric TBI; 25% of all young people with a history of paediatric TBI developed significant levels of externalizing behaviours [36]. Our analyses demonstrating that prisoners with ADHD suffer a high frequency of head injuries before the age of 16 may be explained by the possible coexistence of CD. This may suggest that traumatic events before adulthood may be driven by behavioural problems coexisting with ADHD.

Our results are also consistent with reports from large cohort studies at the population level. Researchers have found evidence of a prospective association between head injury before age 2 and a later diagnosis of ADHD [20]. Nonetheless, they did not find robust evidence of a causal link, as there was also a similar increase in the probability of ADHD diagnosis amongst children who had suffered non-head injuries. They concluded that any accident before age 2 could be a marker for a later diagnosis of ADHD [6], concurring with the view that ADHD is a risk factor for accidents and ensuing injuries. Early brain injury may interact with other predisposing factors generating a “double-burden” on psychiatric disease. Further population-based research should aim to elucidate and expand potential mechanisms.

Prisoners in our study without ADHD or likely TBI had an average QALY of 0.72, which was notably lower than estimates for the general population. Using the HUI-3 and EQ-5D, general population norms in the US and UK estimate an average age-relevant QALY of 0.93 and 0.909, respectively [25, 37]. We analysed the impact of ADHD and TBI on HRQoL and QALYs. We calculated that adult prisoners with ADHD and ADHD with co-morbid TBI have an adjusted average QALY loss of 0.20 and 0.30 compared with those with neither. Prisoners with TBI-only showed no reduction in QALYs. Although the linear combination of coefficients for ADHD-only versus ADHD with co-morbid TBI was not statistically significant, the 0.10 point difference indicated clinical relevance according to the 0.03 difference threshold advised by the HUI3 developers [26]. Furthermore, prisoners with ADHD with co-morbid TBI had a remarkable and significant difference from the TBI-only group, which shows the extent of health impairment that may be attributable to ADHD, and its potential additivity when combined with TBI.

Analyses of specific HRQoL attributes demonstrated the types and extent of impairments. Our finding that prisoners with ADHD had significant impairments related to emotion and cognition, were to be expected, and may likely reflect the influence of co-morbid anxiety or mood disorder [9]. Our finding that prisoners with ADHD with co-morbid TBI had additional significant impairments related to vision, ambulation, and pain was also not a surprise given these groups reported related service use. Prisoners with ADHD with co-morbid TBI had the most visits to specialist mental health and addiction services. ADHD is a known risk factor for developing substance dependence, and a meta-analytic prison rate co-morbidity of 74% was recently established [9].

The significantly poorer vision score among the ADHD with TBI group may relate to their reading difficulties. In the present study, out of the 53 participants in the ADHD/TBI group, 22 (41.5%) required assistance with reading the questionnaires in comparison with only 17.5% of the other participants. With respect to mobility, the finding that prisoners with ADHD/TBI have significantly poorer ambulation problems may reflect that prisoners with the combined condition suffer more injuries that hinder their mobility compared with other prisoners. For example, data obtained from the Danish registry has reported that the morbidity rate is nearly three times higher if you have ADHD, and that 77.8% of unnatural deaths are accounted for by accidental injury [10].

To the best of our knowledge, this is the first study addressing the health status of adult prison inmates with ADHD and TBI using the HUI3. A previous study of children with ADHD reported that higher symptom dimensions of inattention, hyperactivity and impulsivity correlated with poorer HRQoL, along with conduct problems, physical complaints, and somatic symptoms [38]. Additionally, a UK cross-sectional study reported that children and adolescents with ADHD had poorer scores in most health domains compared with samples of healthy children and children with diabetes [39], highlighting the negative impact of ADHD on health. The impact of ADHD on HRQoL scores may be dependent on symptom severity and the presence of co-morbid conditions [40]. Our findings therefore provide initial evidence of the effect that ADHD and TBI has on health-related quality of life outcomes of a fairly large number of prison inmates.

### Limitations

A key strength of the study is its large sample size and a methodology in which every participant was clinically diagnosed using the DIVA-2. Nonetheless, there are several limitations.

The cross-sectional design of the study limits any causal interpretation of the association between TBI and ADHD, and other response variables.

Definitions of TBI often vary depending on the source of information, occurrence of LOC and/or loss of memory, and because of this, many results in studies of TBI cannot be generalized. For example: study participants had to rely on their own or a family member’s recollection of their trauma events. Recall bias is unaccounted for and may have been a factor on all reported measures of head injury, health status, and service use.

Another limitation is that our findings may not be applicable and generalized beyond the prison population and will likely require replication using other clinical samples. Furthermore, our study results may not be applicable and generalized to female or ethnic minority groups of prisoners. Females often present as clinically distinct from males in correctional services, as they have been found to have the potential for greater impairment in ADHD symptomatology and substance dependence rates [41].

Finally, there is an absence of data in our study on co-morbid factors associated with ADHD and TBI, such as conduct disorder, anxiety, mood disorders, substance misuse, antisocial behaviours, and aggression [42–46], which may impact the findings regarding quality of life. Therefore, the associations found with regard to ADHD, TBI, and HUI3 may be caused by variables not investigated in the present study.

## Conclusions

Both ADHD and TBI manifest in high proportions across prison samples. We provide evidence of a strong direct association between them, and evidence to suggest that prisoners with ADHD with co-morbid TBI present with significant impairment in several aspects of HRQoL. Prison inmates with ADHD and those with ADHD with co-morbid TBI had reduced 0.20 and 0.30 QALY respectively when compared with inmates with neither.

The comprehensive health screening tool (CHAT) is an effective screen for both ADHD and TBI in youth offender institutions [35]. The brief version of the Barkley Adult ADHD Rating Scale (B-BAARS) is an effective screen for ADHD with excellent sensitivity and specificity in adult prison populations [27]. Routine administration in all secure establishments of validated screens could support identification of ADHD and head injury. Furthermore, managing the short and long-term consequences of TBI in prisoners may be important in supporting their rehabilitation and reducing reoffending.

## Abbreviations

A&E: Accident and emergency services
ADHD: Attention-deficit hyperactivity disorder
BAARS-IV: Barkley Adult ADHD Rating Scale-IV
B-BAARS: Brief version of the Barkley Adult ADHD Rating Scale
CD: Conduct disorder
CHAT: Comprehensive health screening tool
DIVA-2: Diagnostic Interview for ADHD in Adults 2.0
DSM-5: Diagnostic and Statistical Manual of Mental Disorders, 5th edn
HRQoL: Health-related quality of life
HUI3: Health Utilities Index Mark 3
LOC: Loss of consciousness
MCS: Mayo Classification System
OLS: Ordinary least-squares
OR: Odds ratios
QALY: Quality of life adjusted years
TBI: Traumatic brain injury
WASI: Wechsler Abbreviated Scale of Intelligence
